# Evaluation of Potential Mechanisms Controlling the Catalase Expression in Breast Cancer Cells

**DOI:** 10.1155/2018/5351967

**Published:** 2018-01-28

**Authors:** Christophe Glorieux, Juan Marcelo Sandoval, Nicolas Dejeans, Sandrine Nonckreman, Khadija Bahloula, Hélène A. Poirel, Pedro Buc Calderon

**Affiliations:** ^1^Metabolism and Nutrition Research Group, Louvain Drug Research Institute, Université catholique de Louvain, 1200 Brussels, Belgium; ^2^Facultad de Ciencias de la Salud, Universidad Arturo Prat, 1100000 Iquique, Chile; ^3^Centre de Génétique Humaine, Cliniques Universitaires Saint-Luc & de Duve Institute, Université catholique de Louvain, 1200 Brussels, Belgium

## Abstract

Development of cancer cell resistance against prooxidant drugs limits its potential clinical use. MCF-7 breast cancer cells chronically exposed to ascorbate/menadione became resistant (Resox cells) by increasing mainly catalase activity. Since catalase appears as an anticancer target, the elucidation of mechanisms regulating its expression is an important issue. In MCF-7 and Resox cells, karyotype analysis showed that chromosome 11 is not altered compared to healthy mammary epithelial cells. The genomic gain of *catalase* locus observed in MCF-7 and Resox cells cannot explain the differential catalase expression. Since ROS cause DNA lesions, the activation of DNA damage signaling pathways may influence catalase expression. However, none of the related proteins (i.e., p53, ChK) was activated in Resox cells compared to MCF-7. The c-abl kinase may lead to catalase protein degradation via posttranslational modifications, but neither ubiquitination nor phosphorylation of catalase was detected after catalase immunoprecipitation. Catalase mRNA levels did not decrease after actinomycin D treatment in both cell lines. DNMT inhibitor (5-aza-2′-deoxycytidine) increased catalase protein level in MCF-7 and its resistance to prooxidant drugs. In line with our previous report, chromatin remodeling appears as the main regulator of catalase expression in breast cancer after chronic exposure to an oxidative stress.

## 1. Introduction

Catalase mainly catalyzes the dismutation of hydrogen peroxide (H_2_O_2_) into water and molecular oxygen. This antioxidant enzyme is expressed in all major body organs especially in the liver, kidney, and erythrocytes. In these organs, catalase plays an essential role in cell defense against oxidative stress [1, 2]. A decrease in catalase activity is thus frequently associated with several diseases. For instance, some polymorphisms into the promoter or introns of the *catalase* gene are involved in diabetes, hypertension, vitiligo, Alzheimer's disease, and acatalasemia [3, 4]. Interestingly, catalase is also frequently downregulated in tumor tissues compared to normal tissues of the same origin [5–7]. In this context, when compared to their normal healthy counterparts, we have reported a severe decrease of catalase activity in TLT cells, a murine hepatocarcinoma cell line [8]; in K562 cells, a human chronic myeloid leukemia cell line [9]; and in MCF-7 cells, a human breast carcinoma cell line [10]. These observations are consistent with the study of Sun et al., who showed that immortalization and transformation of mouse liver cells with SV40 virus results in a decrease in catalase activity, which contributes to oncogenesis by increasing reactive oxygen species (ROS) level in transformed cells [11]. The mechanisms controlling the transcription of *catalase* gene are poorly understood, and diverse mechanisms have also been proposed to regulate catalase expression [3].

We explored a potential role of catalase during the acquisition of cancer cell resistance to chemotherapeutic agents. To this end, we overexpressed human catalase in MCF-7 breast cancer cells. No particular resistance against conventional chemotherapies like doxorubicin, cisplatin, and paclitaxel was observed in cells overexpressing catalase, but they were more resistant to prooxidant therapies [12]. Furthermore, we generated a resistant cell line by chronic exposure of MCF-7 cells to an H_2_O_2_-generating system, namely, the ascorbate/menadione (Asc/Men) combination. Catalase was overexpressed in resistant-Resox cells when compared to parental MCF-7 cells [13, 14]. In these cells, transcription factors (i.e., RAR*α* and JunB) and other proteins belonging to coactivator or corepressor complexes (i.e., HDACs) affect chromatin remodelling and lead to the activation or repression of *catalase* gene [10].

Additional regulatory levels clarifying this altered catalase expression in cancer cells were also explored. Since ROS induce DNA lesions, we were interested to know whether a potential role of DNA repair pathways may have an impact on the regulation of catalase expression. Genetic alterations such as loss of heterozygosity or amplification of the *catalase* gene locus, although very rare, were investigated. Both posttranscriptional and posttranslational catalase modifications were also analysed regarding putative alterations of protein stability. Finally, since gene transcription is also regulated by chromatin modulation due to histone acetylation or DNA methylation, these epigenetic marks were also investigated as potential modulators of altered catalase expression in breast cancer cells.

## 2. Materials and Methods

### 2.1. Cell Lines and Chemicals

MCF-7 cells were purchased at ATCC (Manassas, VA, United States). An MCF-7 cell line resistant to oxidative stress (namely Resox cells) was generated by chronic exposure of cells to increasing concentrations of the prooxidant combination of ascorbate/menadione (Asc/Men) for 6 months, starting with 0.5 mM ascorbate/5 *μ*M menadione to a final concentration of 1.5 mM ascorbate/15 *μ*M menadione. Cells were first treated at 50% confluence by replacing their media with fresh media containing Asc/Men. When surviving cells reached 50% confluence, they were washed with warm PBS and treated again [13]. To avoid the development of islets of resistance, which could arise from cooperation between cells, the cells were trypsinized every 2 weeks and subcultured into new flasks. After selection, the cell line was stabilized in drug-free medium for 1 month. Cells were kept in DMEM medium supplemented with 10% foetal calf serum, in the presence of penicillin (100 U/ml) and streptomycin (100 *μ*g/ml) from Gibco (Grand Island, NY, USA). Human mammary epithelial cells 250MK were provided by Dr. M. Stampfer and Dr. J.C. Garbe (Lawrence Berkeley National Laboratory, Berkeley, California, USA). They were maintained in a M87A + CT + X medium and used between passages 8 and 10 [15].

Sodium ascorbate, menadione sodium bisulfite, MG132, actinomycin D, and 5-aza-2′-deoxycytidine were purchased from Sigma (St. Louis, MO, USA).

### 2.2. Conventional Cytogenetic Analysis

Metaphase chromosomes were obtained according to standard protocols from the different cell lines [16]. Briefly, cultured cells, in exponential growth phase, were treated for 4 h with 0.02 *μ*g/ml of Colcemid (Invitrogen). Harvested cells from the flasks after trypsinization were incubated for 30 minutes at 37°C in hypotonic 0.055 M KCl and fixed in a 3 : 1 methanol : glacial acetic acid solution. Chromosome harvesting and metaphase slide preparation were performed according to standard procedures [16–18]. Twenty Reverse Trypsin Wright (RTW) banded metaphases were analysed and karyotypes were reported according to the last 2013 International System for Human Cytogenetics Nomenclature (ISCN 2013).

### 2.3. Fluorescence In Situ Hybridization (FISH)

Specific BAC RP11-964L11 (catalase/11p13; stained with Cy3: red spots) and RP11-90K17 (control/11q14; stained with FITC: green spots) probes from the UCSC (http://genome.ucsc.edu) databases were obtained from the BACPAC Resources Centre at the Children's Hospital Oakland Research Institute (Oakland, CA, USA).

The FISH (fluorescence in situ hybridization) assay was carried out on nuclei and metaphases from fixed pellet of cell as previously described [19]. All hybridized metaphases were captured on a Zeiss Axioplan 2 microscope (Zeiss, Zaventem, Belgium) and analysed using the Isis software (Metasystems, Altlussheim, Germany).

### 2.4. Immunoblotting

The procedures for protein sample preparation from cell cultures, protein quantification, immunoblotting, and data analyses were performed as previously described [12, 14]. Antibody against catalase (#AB1212) was obtained from Millipore (Merck KGaA, Darmstadt, Germany); antibodies against c-abl (#sc-23) and p53 (#sc-126) were from Santa Cruz Biotechnology (Santa Cruz, CA, USA); antibodies against phosphocatalase Y385 (#ab59429) and *β*-actin (#ab6276) were from Abcam (Cambridge, UK); antibodies against phospho-Chk2 T68 (#2661) and phospho-p53 S15 (#9284) were from Cell signaling (Beverly, MA, USA); and antibody against Flag (#F3165) was from Sigma (St. Louis, MO, USA). Protein bands were then detected by chemiluminescence, using the ECL detection kit (Pierce, Thermo Scientific, Rockford, IL, USA). When appropriate, bands obtained via Western blot analysis were quantified, using ImageJ software (http://rsb.info.nih.gov/ij/). Protein expression was normalized to that of *β*-actin.

### 2.5. Real-Time PCR

Total RNA was extracted with the TriPure reagent from Roche Applied Science Diagnostics (Mannheim, Germany). Reverse transcription was performed using SuperScript II RNase H- reverse transcriptase and random hexamer primers (Invitrogen, Grand Island, NY, USA). Sybr Green Supermix (BioRad, Hercules, CA, USA) was used for qRT-PCR. All Primers sequences were designed from Sigma (St. Louis, MO, USA) and provided in Table 1. The samples were incubated 5 min at 95°C, 40 cycles of 10 s at 95°C and 30 s at 60°C, and followed by a melting curve. The fluorescence in the samples was measured after each cycle in a Bio-Rad IQ5 thermocycler (Bio-Rad, Hercules, CA, USA). The results were calculated from the following calculation: 2^−(Ct target gene − Ct EF1)^ and matched to the control samples.

### 2.6. Ubiquitination and Phosphorylation Assays

Cells were transiently transfected, at 50% confluence, with 1 *μ*g of plasmid pcDNA3 coding an ubiquitin-Flag fusion protein, a kind gift from Prof. J-B. Demoulin (UCL, Brussels, Belgium). Twenty-four hours posttransfection, cells were treated with 25 *μ*M MG132 for 5 h. Cells were washed twice with ice-cold PBS and then resuspended in the lysis buffer in the presence of proteases (Protease Inhibitor Cocktail, Sigma, St. Louis, MO, USA) and phosphatase inhibitors (Phosphatase Inhibitor Cocktail, Calbiochem, Merck KGaA, Darmstadt, Germany). Cell lysates were immunoprecipitated in columns containing catalase antibody (AB1212, Millipore), using coimmunoprecipitation kit from Pierce (Rockford, IL, USA). Eluates were then tested by immunoblotting.

### 2.7. MTT Assay

The effects of Asc/Men after incubation with DNMT inhibitor on cell metabolic status were assessed by following the reduction of MTT (3-(4,5-dimethylthiazolyl-2)-2,5-diphenyltetrazolium bromide) to blue formazan [20]. Blue formazan crystals were solubilized with DMSO and the coloured solution was subsequently read at 550 nm. Results are expressed as % of MTT reduction compared to untreated control conditions.

### 2.8. Statistics

All experiments were performed at least in triplicates. Groups were analysed using unpaired *t*-test performed with GraphPad Prism software (San Diego, CA, USA). The level of significance was set at *p* < 0.05.

## 3. Results and Discussion

### 3.1. Is a Genomic Gain of *Catalase* Locus in Breast Cancer Cell Lines Responsible for Catalase Overexpression in Resistant Cells?

**Figure 1:**
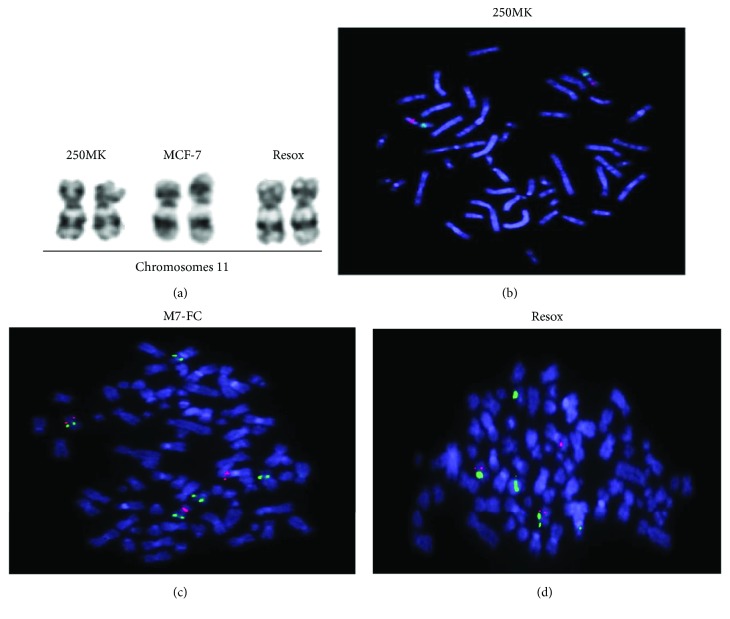
Genomic gain of *catalase* locus in breast cancer cell lines is not responsible for catalase overexpression in resistant cells. (a) Karyotypes: chromosome 11 of human normal epithelial cells (250MK) and breast cancer cell lines. (b) Hybridization of catalase (red spots) and control (green spots) FISH probes on metaphases of 250 MK cells. (c) MCF-7 and (d) Resox cells.

Since the human *catalase* gene is located on the short arm of chromosome 11 (11p13) [21] and deletion of this chromosomal region is generally associated with a decrease of catalase activity, we first focused on genetic alterations, an important hallmark of cancer. Interestingly, the deletion of chromosome 11p is frequently observed in later passages of SV40-transformed human fibroblasts and correlated with a low catalase activity [22]. Such alteration may occur in children affected by WAGR syndrome, a rare genetic disease in which the affected children are predisposed to develop Wilms' tumor (tumor of the kidney), aniridia (absence of the iris), gonadoblastoma, and mental retardation [23–29]. This chromosomal region was altered in the breast cancer cell lines, but karyotypes showed that chromosome 11 was not altered in MCF-7 and Resox cells compared to healthy 250MK, a human epithelial mammary cell line (Figure 1(a)). The complete karyotype analyses of these three cell lines have been previously published [13].

Loss of alleles (i.e., loss of heterozygosity) of *catalase* gene was also observed in non-small-cell lung cancer and was associated with a decrease of catalase activity [30–32]. On the contrary, gain of *catalase* gene copy number and amplification of chromosome 11p can also explain an increased expression of catalase. This phenomenon has been observed in HL-60 cell lines rendered resistant to H_2_O_2_. These cells were more resistant because they have an enhanced catalase activity that correlated with an increase of gene copy number from two to eight times higher than in parental cell line [33].

We have thus investigated a potential loss of heterozygosity or a genomic gain by performing hybridization of catalase and control FISH probes on metaphases. The catalase and control FISH probes were controlled in human lymphocytes (data not shown) and normal mammary epithelial 250MK cells (Figure 1(b)). We observed two red (*catalase* locus) and two green spots (control) localized on the two chromosome 11. A genomic gain of *catalase* locus was observed in MCF-7 and Resox cell lines compared to 250MK cells: we counted 3 *catalase* spots, two localized on the two normal chromosome 11 and one localized on an unidentified chromosome (Figures 1(c) and 1(d)). However, the number of *catalase* loci remained similar in these two cell lines. For Resox cells, one subclonal population was characterized by 6 red spots and two-fold chromosome number (not shown) corresponding to 20% of total Resox population. We can conclude that this genomic gain of chromosome 11p13 is not involved in mechanisms leading to catalase overexpression in Resox cells.

### 3.2. Are Proteins of DNA Damage Pathway Involved in Catalase Expression of Breast Cancer Cells?

Since the karyotypes were altered in breast cancer cell lines [13] and ROS induce DNA damage, we investigated whether the activation of DNA repair system may influence the regulation of catalase expression. To our knowledge, a putative link between this repair system and antioxidant enzyme expression as a possible response against ROS-mediated DNA damage has not yet been investigated.

**Figure 2:**
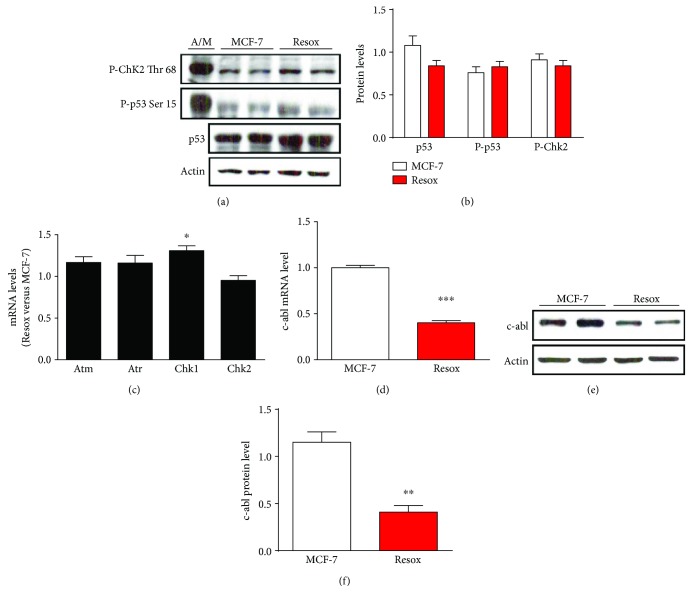
DNA damage pathway did not enhance catalase expression in breast cancer cell lines. (a and b) Immunoblotting and protein quantification of p53, P-p53, and P-Chk2 in breast cancer cells. Ascorbate 1 mM/menadione 10 *μ*M (A/M) for 2 h is used as a positive control to induce DNA damage. (c) mRNA levels of the different kinases activated during the DNA damage pathway. (d) c-abl mRNA level in both MCF-7 and Resox cells. (e and f) Immunoblotting and protein quantification of c-abl in breast cancer cells. Data are mean ± s.e.m. Groups were compared using unpaired *t*-test. ^∗^ *p* value <0.05; ^∗∗^ *p* value <0.01; ^∗∗∗^ *p* value <0.001.

Three different kinases, namely, DNA-PK (DNA-activated protein kinase), ATM (ataxia telangiectasia mutated), and ATR (ataxia telangiectasia and Rad3 related), are activated when the DNA is damaged leading to the activation of proteins involved in DNA repair [34, 35]. These pathways induced a cascade of protein kinases such as Chk1 and Chk2 (checkpoint kinases), which activate protein p53 inducing *γ*-histone H2AX phosphorylation. Neither p53 nor ChK2 proteins appeared activated in Resox cells (Figures 2(a) and 2(b)), whereas a strong activation was observed in control MCF-7 cells incubated with Asc/Men (Figure 2(a)). Moreover, among the mRNA levels of the different kinases involved in the signaling cascade, only a slight increase of Chk1 mRNA level was observed in Resox cells (Figure 2(c)).

The protein c-abl (Abelson murine leukemia viral oncogene homolog 1) is also induced during the activation of DNA damage pathway [36]. Cao et al. demonstrated that c-abl is capable of phosphorylating catalase leading to its subsequent ubiquitination and degradation by the proteasome [37, 38]. Interestingly, c-abl mRNA and protein levels were decreased in Resox cells compared to MCF-7 cells (Figures 2(d)–2(f)).

Altogether, these results show that DNA damage pathway is not the cause of catalase overexpression in Resox cells, but posttranslational modifications mediated by c-abl might occur on catalase protein.

### 3.3. Are Catalase Covalent Modifications Occurring in Breast Cancer Cells?

Various posttranslational modifications such as phosphorylation, ubiquitination, acetylation, glycosylation, and covalent binding with other proteins (i.e., p53, Atm) have been reported to modulate both catalase expression and activity at different levels [37–43]. The half-life of catalase is generally high, reaching more than 3 days [44–46]. When cells are under oxidative stress conditions, c-abl and c-abl-related gene (Arg) tyrosine kinases are able to phosphorylate catalase at Tyr231 and Tyr386 [37, 38]. The phosphorylated enzyme is subsequently ubiquitinated and degraded by the proteasome. It appears that a physical interaction exists between these kinases and catalase, as demonstrated by immunoprecipitation assays performed in cancer cells and KO MEF (mouse embryonic fibroblast) for the kinases. Furthermore, two proteasome inhibitors, namely, MG132 and lactacystin, also restored catalase expression in these cells [37, 38].

**Figure 3:**
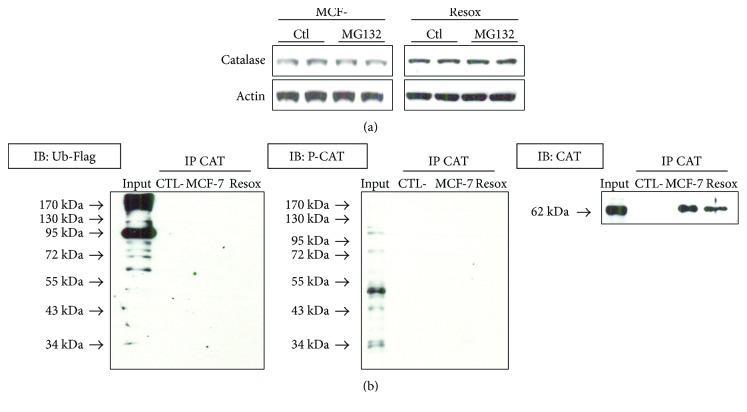
Catalase is neither phosphorylated nor ubiquitinated in breast cancer cells. (a) Catalase protein levels were measured after 5 h incubation with proteasome inhibitor MG132 in both MCF-7 and Resox cells. (b) Immunoprecipitation (IP) with anticatalase antibody and immunoblotting (IB) with anti-Flag, antiphosphocatalase, and catalase antibodies. Prior IP, cells were transfected with a plasmid pcDNA3 (1 *μ*g) coding an ubiquitin-Flag for 72 h.

Catalase posttranslational modifications were analysed in our breast cancer cells model. The MG132 proteasome inhibitor did not modify catalase expression in MCF-7 and Resox cells (Figure 3(a)). Finally, catalase phosphorylation did not appear to play a role as a main covalent modification. Indeed, neither ubiquitination nor phosphorylation of catalase was detected after catalase immunoprecipitation (Figure 3(b)), although c-abl protein level was decreased in Resox cell line (Figure 2(e)).

### 3.4. Are Posttranscriptional Modifications Playing a Role in Catalase Overexpression in Resox Cells?

The expression of catalase expression can also be regulated at the RNA level. The *catalase* gene possesses a 3′ flanking region with T-rich clusters and CA repeats that are susceptible to be regulated by some redox-sensitive proteins, which bind catalase mRNA and enhances translation [47]. Some unidentified proteins could bind to the 5′UTR (untranslated region) of the catalase mRNA to accelerate the transcriptional rate, as has been observed in cancer PC12 cells exposed to H_2_O_2_ [48].

The microRNA miR-451 can also modulate and enhance catalase expression by suppressing the protein 14-3-3*ξ*, an inhibitor of the FoxO3a (forkhead box O3a) pathway, thereby protecting cells against oxidant drugs [49], but this transcription factor appears to not play a critical role in our models [14]. Moreover, microRNA miR-30b can bind directly to the 3′UTR region of the catalase mRNA, on a conserved site across several species. Mimics of miR-30b can drastically decrease the catalase protein level in human retinal pigment epithelial cell ARPE-19 [50].

**Figure 4:**
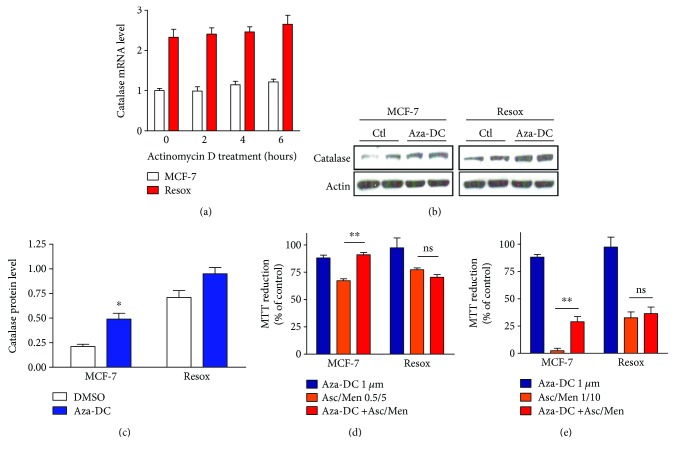
Evaluation of mRNA stability in MCF-7 and Resox cells. DNA methyltransferase inhibitor 5-aza-2′-deoxycytidine tends to increase the levels of catalase protein level in MCF-7 cells. (a) Catalase mRNA levels were measured in MCF-7 and Resox cells after 2, 4, and 6 h of actinomycin D treatment (10 *μ*g/ml). (b and c) Immunoblotting and protein quantification of catalase, after 72 h incubation with 1 *μ*M of 5-aza-2′-deoxycytidine (Aza-DC). (d and e) Cells were prior incubated with 1 *μ*M of 5-aza-2′-deoxycytidine for 72 h, then with various concentrations of ascorbate (Asc, mM) and menadione (Men, *μ*M). Cell survival was measured by MTT assay. Data are mean ± s.e.m. Groups were compared using unpaired *t*-test. ^∗^ *p* value <0.05; ^∗∗^ *p* value <0.01.

In a MCF-7 cell line rendered resistant to H_2_O_2_ and as a result overexpressed catalase, a treatment with actinomycin D (an inhibitor of the transcription) induces a delay in the degradation of catalase mRNA [51]. As previously shown, Resox cells have more mRNA than MCF-7 suggesting that mRNA stability in the former cells might be higher than in MCF-7 cells [10]. However, the levels of catalase mRNA did not decrease after 6 hours of actinomycin D incubation in both cells (Figure 4(a)).

### 3.5. DNA Methyltransferase Inhibitor Increases Catalase Protein Level in MCF-7 Cells

Since the transcription of a gene can be regulated by chromatin remodelling due to histone acetylation or DNA methylation, the importance of these mechanisms for the expression of catalase in cancer cells has been raised. In this context, epigenetic changes were proposed to regulate catalase expression in acute myelogenous leukaemia resistant to doxorubicin (AML-2/DX100 cells), which exhibit less catalase activity compared to their parental cell lines [52]. Indeed, both Trichostatin A (TSA), an inhibitor of histone deacetylases (HDAC), and 5-aza-2′-deoxycytidine, an inhibitor of DNA methylation, increase the protein levels of catalase in the AML-2/DX100 cells. These data were then confirmed by chromatin immunoprecipitation and sodium bisulfite sequencing assays. The results suggested a hypoacetylation of histones H4 but not H3. DNA hypermethylation on the catalase promoter in AML-2/DX100 cells was also observed [52]. We have also described the acetylation of histones (at least histone H4), leading to an opening repression of chromatin structures near −1518/−1201 promoter region in MCF-7 cells [10], as shown by ChIP (chromatin precipitation) assays.

DNA methylation is also involved in the regulation of catalase. Indeed, specific CpG islands in the human promoters of *catalase* and *Oct-1* (octamer-binding transcription factor 1: an inducer of *catalase* gene transcription) genes were methylated in human hepatocellular carcinoma cell line after H_2_O_2_ treatment. This is in good correlation with a decreasing catalase expression in this model [53, 54]. In this particular type of cancer, the catalase promoter is hypermethylated in the tumor itself but not in the neighbouring tissues [55]. On the contrary, DNA hypomethylation of the *catalase* gene is frequently observed in colon tumors whereas few modifications of DNA methylation are observed in breast adenocarcinoma compared to normal breast tissues. In the studies where a change in the DNA methylation pattern was observed, DNA hypomethylation and hypermethylation occurred only around the exon 2 of the *catalase* gene [55, 56].

We have thus explored the possibility that DNA methylation may regulate catalase expression in our cellular models. The DNA methyltransferase (DNMT) inhibitor, 5-aza-2′-deoxycytidine, tends to increase catalase protein level in MCF-7 and not significantly in Resox cells (Figures 4(b) and 4(c)). Moreover, MCF-7 cells became more resistant to prooxidant drugs (Asc/Men) after preincubation with the DNMT inhibitor (Figures 4(d) and 4(e)). Once again, our results demonstrate a potential role of chromatin remodelling in the regulation of catalase expression during cancer cell resistance acquisition to a chronic prooxidant treatment. Consistent with our previous study, methyl CpG-binding proteins (i.e., MECP2) and DNMT (i.e., DNMT1) were identified by AP-MS (affinity purification followed by mass spectrometry) analyses and interact with −1518/−1200 promoter region of *catalase* gene in MCF-7 cells [10]. Altogether, it suggests that DNA methylation and posttranslational modifications of histones are events associated to repress the transcription of *catalase* gene. In a panel of 12 cancer cell lines treated with TSA, we confirmed that chromatin remodelling is not a general regulatory mechanism in cancer cells but another breast cancer cell line, an estrogen receptor-negative MDA-MB-231 cell line, also showed increased catalase protein level when incubated with HDAC or RAR*α* (retinoic acid receptor alpha) inhibitors [10]. Moreover, RAR*α* overexpression decreased drastically the expression of catalase and histone acetylation was not detected on the catalase promoter by ChIP assay in MDA-MB-231 cells [data not shown], suggesting a similar mechanism regulating catalase expression in these breast cancer cells. In this context, we have recently reported that targeting the redox status of cancer cells by modulating catalase expression is emerging as a novel approach to potentiate chemotherapy [57]. Modifying epigenetic changes such as histone acetylation or DNA methylation in breast cancer cells will thus dramatically alter the expression of various genes including catalase, thus sensitizing cells to prooxidant chemotherapies [3, 10, 57].

## 4. Final Concluding Remarks

Regulatory mechanisms involved in catalase expression occur at different levels from genetic to posttranscriptional modifications including epigenetic and transcriptional processes [3]. Loss of heterozygosity or gene amplification may contribute to an altered expression of catalase but data obtained in this study indicate a minor role as compared to other regulatory mechanisms such as *catalase* gene transcription as we have recently reported [10, 14]. Meanwhile, although ROS induce DNA damage, the activation of the DNA repair system did not lead to a conclusive role of these pathways on regulation of catalase expression either in MCF-7 or Resox cells. Neither posttranscriptional nor posttranslational modifications are likely involved in the different catalase protein levels in both cell lines. Instead, we have already shown that histones H4 were acetylated around the promoter region −1518/+16 in Resox cells, explaining the catalase overexpression in these cells. In this study, we have demonstrated that DNA hypo/hypermethylation also plays a pivotal role in this regulation and resistance to prooxidant drugs.

Since catalase expression is sensitive to redox modulation, a therapeutic strategy may be developed in the context of cancer, taking in mind the level of catalase expression. For instance, if catalase is downregulated and the clinical option is to get increased amounts of catalase, an interesting approach would be the use of epigenetic agents (DNMT or HDAC inhibitors) in order to promote a change in chromatin remodelling. Another possibility would be the use of antagonists of RAR*α*, leading to a pharmacological inhibition of this nuclear receptor and consequently to a loss of catalase repression. Indeed, we recently showed that both compounds, TSA and Ro 41-5255 (HDAC and RAR*α* inhibitor, resp.), enhance catalase expression not only in MCF-7 cells but also, as previously mentioned, in other mammary cell lines such as the highly aggressive and metastatic MDA-MB-231 cells [10]. Conversely, in case of catalase overexpression, a therapeutic option would be the use of siRNA against catalase coupled to nanoparticles or, as we have recently shown, the use of arsenic trioxide (Trisenox) which decreases catalase expression likely by activating the Akt/PKB (protein kinase B) signalling pathway and/or inducing the expression of RAR*α* [57, unpublished results]. It should be noted that the hypothesis suggesting that chromatin remodelling as the main event controlling catalase expression requires both activating and inhibitory factors. Indeed, we have recently shown that the transcription factors JunB and RAR*α* are involved in the positive and negative expression of catalase by recruiting coactivators and corepressors leading to chromatin remodelling [10].

Taking together, these findings and previous results obtained in our laboratory [10, 12, 14, 57] suggest that chromatin remodelling is a major regulatory process controlling catalase expression in breast cancer cells during resistance acquisition against an oxidative stress.

## Abbreviations

Akt/PKB: Protein kinase B
AP-MS: Affinity purification followed by mass spectrometry
Arg: c-abl-related gene
ATM: Ataxia telangiectasia mutated
ATR: Ataxia telangiectasia and Rad3 related
Asc/Men: Ascorbate/menadione
c-abl: Abelson murine leukemia viral oncogene homolog 1
ChIP: Chromatin precipitation
Chk: Checkpoint kinase
DNA-PK: DNA-activated protein kinase
DNMT: DNA methyltransferase
FISH: Fluorescence in situ hybridization
FoxO3: Forkhead box O3
HDAC: Histone deacetylase
MEF: Mouse embryonic fibroblast
Oct-1: Octamer-binding transcription factor 1
RAR*α*: Retinoic acid receptor alpha
ROS: Reactive oxygen species
TSA: Trichostatin A
UTR: Untranslated region
WAGR: Wilms' tumor
Aniridia: Gonadoblastoma and mental retardation (syndrome).

## Supplementary Materials

Supplementary Materials Table S1: Oligonucleotides (qRT-PCR).
