# Supplementary material for: Evaluation of Potential Mechanisms Controlling the Catalase Expression in Breast Cancer Cells
Source: Oxid Med Cell Longev. 2018 Jan 28;2018:5351967. doi: 10.1155/2018/5351967 (PMC5829333; doi:10.1155/2018/5351967)
Supplement: Supplementary Materials — Table S1: Oligonucleotides (qRT-PCR). [file 5351967.f1.pdf]

Table S1. Oligonucleotides (qRT-PCR).

Gene	Forward primer	Reverse primer
Catalase	ccagaagaaagcggtcaagaa	gagatccggactgcacaaag
EF1 (reference)	cttcactgctcaggtgat	gccgtgtggcaatccaat
c-ABL	aaaaccttctcgctggaccc	tttgggcttcacaccattcc
ATM	ccgactttgttccctctggctt	cctaggcctcccatcatctt
ATR	tgaagacttggtttacctcc	tgcatactcatcaactgcaaa
Chk1	ctcaagaaaggggcaaaaag	ggttctggctgagaactgga
Chk2	ggagggacaaaagctgtgaa	tccctgaaaatccgaaagtg
